# Generating Aptamers by Cell-SELEX for Applications in Molecular Medicine

**DOI:** 10.3390/ijms13033341

**Published:** 2012-03-12

**Authors:** Mao Ye, Jun Hu, Minyuan Peng, Jing Liu, Jun Liu, Huixia Liu, Xielan Zhao, Weihong Tan

**Affiliations:** 1 State Key Laboratory of Chem/Biosensing and Chemometrics, College of Biology, Hunan University, Changsha 410082, China; 2 Hunan Provincial Tumor Hospital, Changsha 410013, China; E-Mail: finger3030@163.com; 3 Xiangya School of Medicine, Xiangya Hospital, Central South University, Changsha 410008, China; E-Mails: pengminyuan@yahoo.com.cn (M.P.); jingliucsu@hotmail.com (Ji.L.); yqg979@163.com (Ju.L.); huixialiu@126.com (H.L.); xielanzhao@yahoo.com.cn (X.Z.); 4 Department of Chemistry and Department of Physiology and Functional Genomics, Center for Research at the Bio/Nano Interface, Shands Cancer Center, UF Genetics Institute and McKnight Brain Institute, University of Florida, Gainesville, FL 32611, USA

**Keywords:** aptamer, SELEX, molecular medicine

## Abstract

Aptamers are single-stranded oligonucleotides of DNA or RNA that bind to target molecules with high affinity and specificity. Typically, aptamers are generated by an iterative selection process, called systematic evolution of ligands by exponential enrichment (SELEX). Recent advancements in SELEX technology have extended aptamer selection from comparatively simple mixtures of purified proteins to whole living cells, and now cell-based SELEX (or cell-SELEX) can isolate aptamers that bind to specific target cells. Combined with nanotechnology, microchips, microfluidic devices, RNAi and other advanced technologies, cell-SELEX represents an integrated platform providing ultrasensitive and highly specific tools for clinical medicine. In this review, we describe the recent progress made in the application of cell-SELEX for diagnosis, therapy and biomarker discovery.

## 1. Introduction

Aptamers are single-stranded RNA or DNA sequences that bind to target molecules with high affinity and specificity. Aptamer molecules exist in nature in the form of genetic regulators called riboswitches [1], but artificial aptamers can be obtained by an *in vitro* selection process known as systematic evolution of ligands by exponential enrichment (SELEX), first described by two independent laboratories in 1990 [2,3]. The SELEX process starts with a random pool of 10^13^–10^16^ ssDNA or ssRNA molecules subjected to iterative rounds that specifically enrich sequences having high binding affinity to the target molecules. *In vitro* SELEX has been widely used for the identification of a variety of targets, ranging from small molecules (metal ions, organic dyes, amino acids, or short peptides) to large proteins or complex targets (whole cells, viruses, virus-infected cells, or bacteria). The ability of aptamers to selectively bind to different targets is based on their distinct three-dimensional structure, allowing them to form stable and specific complexes with different targets of complementary shape [4,5]. Thus, in view of target inhibition, aptamers are different from ribozymes and antisense oligonucleotides, which are used to prevent the translation of genetic information from mRNAs to proteins [6]. The binding affinity of aptamers to their targets is very high, with typical dissociation constants in the picomolar to nanomolar range, depending on the nature of the targets. Also, aptamers recognize their targets with extremely high specificity. For example, aptamers can discriminate among homologous proteins that contain only a few amino acid changes [7–9].

The molecular recognition properties of aptamers, such as high affinity and specificity, are similar to antibodies, but the unique properties of aptamers set them apart from antibodies. Aptamers are produced by chemical synthesis rather than tedious biological expression. This allows researchers to quickly and reproducibly synthesize any DNA or RNA sequence with little or no batch-to-batch variation. As synthetic molecules, aptamers readily support site-specific modifications toward a specific purpose. For research, aptamers can be easily labeled with florescent dyes, biotin or radionuclides. For clinical purposes, aptamers can be conjugated to nanoparticles [10], drug molecules [11], enzymes [12], viruses [13] or small interfering RNAs (siRNAs) [14]. Unlike antibodies, aptamers are very stable across a wide range of temperature or storage conditions. Even thermally-denatured aptamers can return to their original conformation without losing binding affinity by one cycle of heating and cooling, whereas antibodies are temperature sensitive and denaturation is usually irreversible. In addition, chemical modifications, such as 2′-fluoro and 2′-*O*-methyl substitutions, can enhance their biochemical stability against nuclease degradation [4,15]. Furthermore, their small size allows for rapid penetration into tissues and organs, with low toxicity and low immunogenicity, which may facilitate long-term therapeutic efficacy and safety. These unique biochemical properties make aptamers highly suitable for the detection, diagnosis and treatment of disease.

## 2. Cell-SELEX

Typically, aptamers are selected by performing *in vitro* SELEX against a purified protein. Using purified proteins as targets has the advantage of easily achieving specific enrichment during the selection process if the target protein assumes a stable conformation. Based on this method, high-affinity aptamers against purified MUC1 peptides [16], the purified extracellular domain of prostate-specific membrane antigen (PSMA) [17], cell adhesion molecule P-selectin [18] and protein tyrosine phosphatase 1B (PTP1B) [19] have been isolated. When target proteins are present in a modified state, however, or if the potential binding domain is masked in a physiological context, the isolated aptamers might not recognize the natural structure of some proteins. Liu *et al.* selected RNA aptamers against the histidine-tagged EGFRvIII ectodomain produced by the *Escherichia coli* expression system [20]. Although generated aptamers exhibited high affinity and specificity for *in vitro* purified protein, it did not bind the full-length EGFRvIII protein expressed on the surface of eukaryotic cells, probably because a post-translational modification altered the structure of EGFRvIII.

To avoid the disadvantage of selected aptamers against a non-native protein conformation, a strategy using whole living cells as targets for aptamer selection, termed cell-SELEX, has been developed. Unlike protein-based SELEX, cell-SELEX does not require any prior knowledge of the protein conformation. Also, it is unnecessary to purify target proteins by processes that may disrupt the native conformation. All cell-surface molecules will remain in their native environment, retain native folding structure and contain possible post-translational modifications throughout the selection process. Thus, aptamers selected using whole live cells will be able to bind to the natural folded conformation of the target on cells. This shows great potential in biomedical research and in the development of cell-specific diagnosis and therapeutics.

To generate aptamers against whole living cells, target and negative cells must first be considered. In general, molecular differences between any two closely-related cell populations, such as tumor cells and normal cells, determine the number of SELEX rounds required and the overall success. To enhance the specificity of the aptamers, negative cells are used in a counter-selection step to avoid the enrichment of aptamers for abundant nonspecific proteins. To monitor enrichment by flow cytometry, the sense strand of the primer is labeled with a fluorophore, whereas sense strands are separated from antisense by conjugating biotin to the reversible primer for streptavidin-biotin interaction [21].

**Figure 1:**
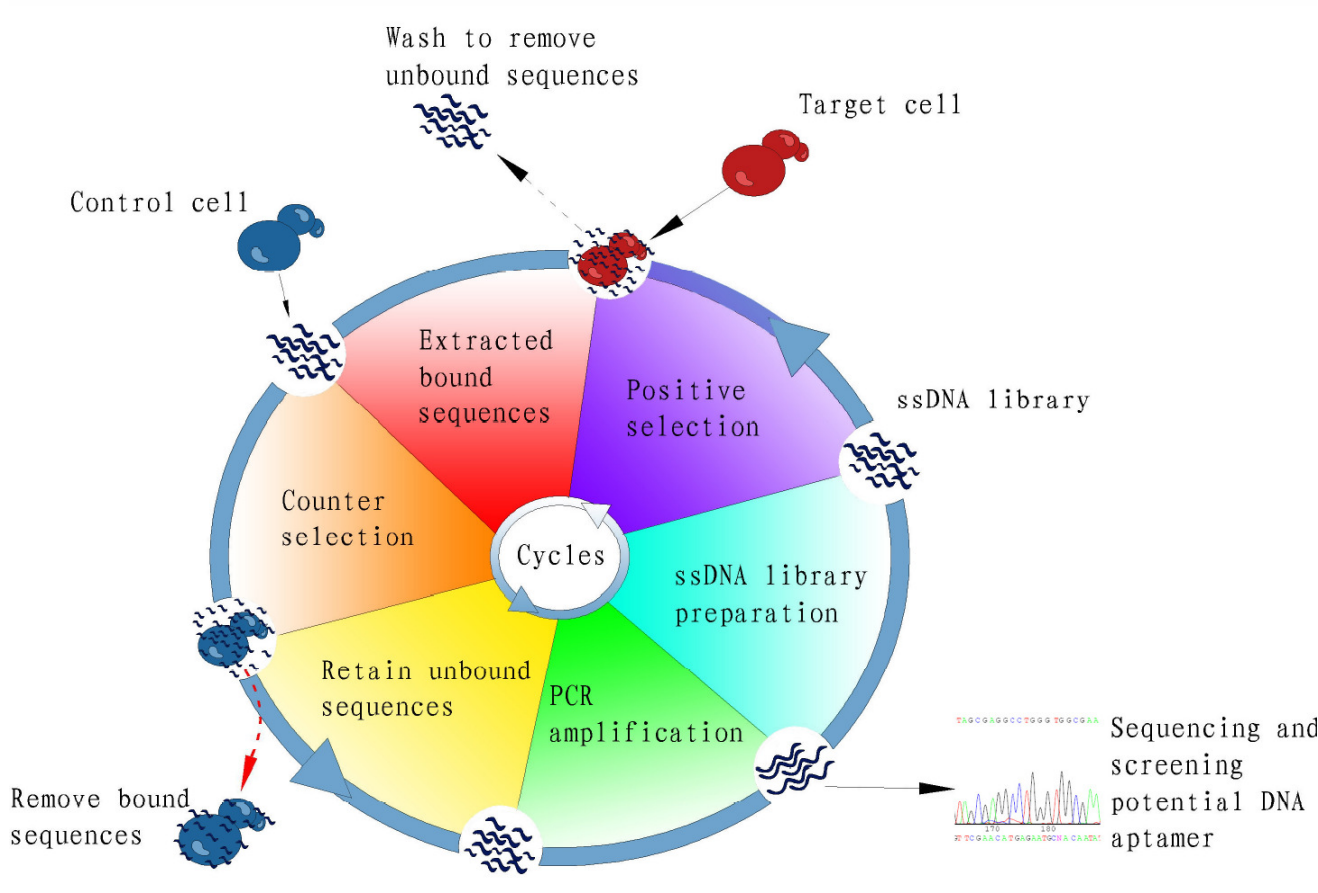
Schematic representation of DNA aptamer selection using the cell-SELEX strategy.

A typical selection cycle for a suspension of cells is shown in Figure 1. Briefly, concentration and viability need to be determined before selection. After serum is removed from culture media by thorough washing, the target cells are incubated with ssDNA on a rotary shaker for a specified time and at a specified temperature, depending on the purpose of selection. Second, unbound aptamers are washed away and bound sequences are eluted by heat denaturation for 15 min at 95 °C. Third, the eluted sequences are amplified by PCR or used for counter-selection. If counter-selection is desired, the negative cells are incubated with the eluate, and the unbound ssDNAs are separated by centrifugation and amplified by PCR. Fourth, the amplified dsDNA products are bound to streptavidin beads. The antisense strand with biotin is retained on the beads, and the sense strand is eluted with NaOH, which does not dissociate the biotin-streptavidin bond. The eluted ssDNAs are the enriched pool from the first round of selection and are then used to construct a library for the next round of selection. In general, the concentration of cells, DNA, fetal bovine serum, ionic strength, and incubation times and temperatures are varied such that each round becomes more stringent, thus favoring the selection of high affinity aptamers.

**Figure 2:**
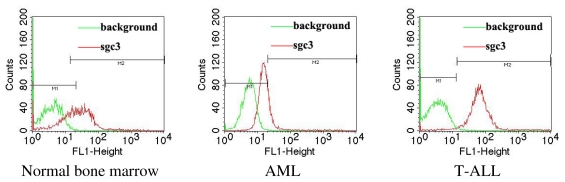
Binding assay of sgc3 in normal bone marrow, AML and T-ALL by flow cytometry.

Since cell-SELEX has the advantage of direct selection of aptamers without previous knowledge of the target molecule, this strategy has been widely adopted to generate aptamers that are capable of recognizing different cell populations, including red blood cells (RBCs) [22], lymphocytic leukemia [23], myeloid leukemia[24], liver cancer [25], small cell lung cancer [26,27], non-small cell lung cancer [28] and ovarian cancer cells [29]. Recently, we extended aptamer selection by cell-SELEX to include clinical samples. By using a human precursor T-cell acute lymphoblastic leukemia (T-ALL) cell line, CCRF-CEM, as the target and the human Burkitt’s lymphoma cell line Ramos as negative cells, we successfully obtained the aptamer sgc3 that specifically recognized cultured leukemia T cells [23]. Furthermore, we applied sgc3 to clinical samples and found that sgc3 exhibited stronger binding affinity to T-lineage acute lymphoblastic leukemia (ALL) samples than to acute myeloid leukemia (AML) and normal bone marrow samples (Figure 2).

## 3. Cell-Specific Aptamers for Biomarker Discovery

Biomarkers indicate a change in the expression or state of proteins or genes under changing physiological conditions or during pathogenesis, thus providing an important tool for clinical diagnosis, monitoring and treatment. The discovery of novel biomarkers not only leads to a greater understanding of disease processes, but also is of great clinical value for early detection and prompt treatment. Although proteomic methods, such as two-dimensional gel electrophoresis (2D-GE) and differential imaging gel electrophoresis (DIGE), followed by mass spectrometric identification of proteins, are employed for new biomarker discovery, the elucidation of membrane proteins that are differentially expressed in disease is still a great challenge [30,31].

Aptamers generated by cell-SELEX facilitate biomarker discovery for membrane protein. As noted above, aptamers can recognize different types of cells, based on molecular difference of unknown membrane proteins on disease cells compared with normal cells. Thus, it is conceivable that new biomarkers could be discovered as long as the aptamer’s target protein can be identified. Following this idea, aptamer-based target membrane protein identification had been developed by affinity purification, followed by sequencing using mass spectrometry. For example, Daniels *et al.* used a glioblastoma-derived cell line, U251, as the target for cell-SELEX. The aptamer GBI-10 was identified and shown to specifically target U251 cells [32]. Furthermore, by using affinity purification and LC-MS/MS analysis, the extracellular matrix protein tenascin-C was determined to be the binding partner of GBI-10. Tenascin-C is a large glycoprotein that is highly expressed in the microenvironment of most solid tumors, which is involved in uncontrolled proliferation, angiogenesis and metastasis of tumor cells [33].

Using cell-SELEX, we developed DNA aptamers that specifically bind to CCRF-CEM cells, a human precursor T-cell acute lymphoblastic leukemia (T-ALL) cell line. In cell-SELEX, a B cell line from human Burkitt’s lymphoma, Ramos, was used as the negative control for counter-selection. After 20 rounds of selection, aptamer sgc8 was isolated and showed high specificity and affinity for CCRF-CEM leukemia cells, while showing no specific binding to lymphoma cells or normal bone marrow cells [34]. To identify target protein of sgc8 for biomarker discovery, aptamer-mediated affinity purification of protein targets was performed. Membrane lysate was incubated with sgc8, labeled with a biotin tag at the 5′-end. The binding complex was then separated using streptavidin-coated magnetic beads. After the captured proteins were eluted by heating and separated by SDS-PAGE, characteristic protein bands on the gel were digested and analyzed using LC-MS/MS QSTAR. Finally, PTK7, a transmembrane receptor tyrosine kinase-like molecule, was successfully identified as a potential biomarker for T-cell acute lymphoblastic leukemia [35].

The same strategy was also adopted for purification and identification of immunoglobulin μ heavy chain (IGHM), a molecular target of aptamer TD05 developed for Ramos cells [36]. To increase the stability of the aptamer-protein complex, TD05 was modified with photoactive 5-dUI to promote covalent cross-linking to the target protein. This modification of TD05 also facilitated the purification and enrichment of the target protein from cell lysate. The IGHM is one of the major components of B-cell receptor complex expressed in mature Burkitt’s lymphoma cells, and IGHM expression on premature B-lymphocytes is closely related to the development of Burkitt’s lymphoma. Thus, cell-SELEX not only provides excellent tools for identifying target proteins, it is also a promising strategy for the discovery of new disease-related biomarkers. Some examples of target identification mediated by cell-SELEX are summarized in Table 1.

## 4. Cell-Specific Aptamers for Targeted Cancer Therapy

Continued progress in molecular medicine, particularly drugs targeting disease-related cells or proteins, will lead to cancer treatment with greater efficacy and lower off-target toxicity. Since cell-binding aptamers possess excellent targeting properties, they hold a great potential for targeted cancer therapy as a therapeutic or delivery agent.

As mentioned above, aptamers generated by cell-SELEX are able to selectively bind disease-related proteins on the cell surface. Therefore, the development of these aptamers as therapeutic agents is based on inhibitory activity against their protein targets that are dysregulated in cancer. For example, RET (rearranged during transfection) is a receptor tyrosine kinase activated by the glial cell line-derived neurotrophic factor (GDNF) family. Mutations of RET result in constitutive activation that causes endocrine neoplasia (MEN) type 2A and 2B syndromes. Certria *et al.* adopted a cell-SELEX strategy to select aptamers against the mutated form of the human RET receptor [39]. Pheochromocytoma-derived PC12 cells overexpressing the mutant extracellular domain of RET receptor were used for positive selection, while two counter-selection steps were employed against parental PC12 cells and PC12 cells overexpressing the wild type extracellular domain of the human RET receptor. After 15 rounds of selection, the resulting aptamers not only specifically bound to mutant RET, but also inhibited RET activation and effectively blocked the RET-mediated signal transduction pathway, which exhibited significant therapeutic potential against the endocrine neoplasia syndrome. Recent cell-SELEX selection procedures have succeeded in producing anticancer aptamers against tenascin-C [32] and TGF-β type III receptor [38].

In some cases, identification of the cell-surface target is not absolutely necessary when using cell-SELEX to generate functional aptamers against cancer. Zueva *et al.* developed aptamers for the specific recognition of highly metastatic cells [40]. Two malignant isogenic hamster cell lines, HET-SR-1 (HM) and HET-SR (LM), were used for cell-SELEX. Although they are similar in many respects, including tumorigenicity and growth properties, the former shows greater metastatic potential *in vivo* than the latter. By using HM for positive selection and LM for counter-selection, aptamers E10 and E37 were identified that specifically bind to the highly metastatic cell line HM. More importantly, the aptamers selected by this process disrupted migration and invasion of tumor cells by decreasing the phosphorylation of multiple metastasis-associated tyrosine kinases. In another study using cell-SELEX, Certria’s group generated aptamers able to discriminate between two highly related phenotypes within the same tumor [41]. The malignant human glioma cell line U87MG was used as the target for the selection step and a similar, but poorly tumorigenic human glioma cell line, T98G, was used for the counter-selection step. They obtained a panel of aptamers capable of binding to target cells with high affinity. Interestingly, these aptamers inhibited a specific intracellular signal transduction pathway and showed functional activity against tumor cell proliferation.

Recently, Boltz and co-workers adopted a strategy that combined two DNA aptamers simultaneously binding to tumor cell and nature killer cells to mediate specific tumor cell lysis [42]. One of the aptamers was generated against CD16α expressed on NK cells, which plays a pivotal role in antibody-dependent cellular cytotoxicity (ADCC). The other aptamer was used to bind to the hepatocyte growth factor receptor (c-Met), usually overexpressed in tumor cells. c-Met is a transmembrane receptor tyrosine kinase, which is involved in the proliferation, migration and invasion of many cancer types [43]. Two aptamers were connected by an ologonucleotide linker as bi-specific aptamers, which can mimic ADCC by recruitment of NK cells via CD16α to c-Met positive tumor cells and subsequently specifically killed tumor cells.

Cell type-specific aptamers that target cancer cell surface receptors have also been exploited as carriers for the delivery of a variety of anticancer substances to given cancer cells. Once delivered, target-specific aptamers selectively increase the accumulation or retention of therapeutic agents in cancer cells but spare noncancerous cells in the tissue. Therefore, such aptamers can enhance the therapeutic efficiency and decrease unwanted side effects of conventional chemotherapeutics, such as the death of normal cells.

Prostate specific membrane antigen (PSMA) is a well-known prostate cancer tumor marker which is overexpressed on prostate cancer epithelial cells [44]. Studies has shown PSMA can be constitutively endocytosed into cells via clathrin-coated pits [45]. Therefore, a strategy to develop PSMA-specific aptamers may serve as a poteinal drug delivery vehicles for prostate cancer. In 2002, Lupold *et al.* identified two 2′-fluoro-pyrimidine (2'Fy)-RNA aptamers that bind to prostate cancer cells via the extracellular domain of PSMA [17]. Subsequently, these aptamers have been employed to deliver chemotherapeutic agents [46–48], toxin [49], or siRNA [14,50,51] for targeted prostate cancer therapy.

Similarly, aptamer sgc8 specifically binding to acute lymphoblastic leukemia (ALL) T-cells via PTK7 on the cell membrane can also be internalized into cells [52]. To develop sgc8 as delivery vehicles for targeted therapy in leukemia, we covalently conjugated doxorubicin (DOX) to sgc8c which is an optimized and truncated DNA sequence of sgc8 and had identical binding properties as sgc8 [53,54]. DOX is an anthracycline-derived drug molecule used to treat many types of cancer, including acute lymphoblastic, myelocytic leukemia and malignant lymphomas. However, application is limited in clinical practice by high toxicity to non-target cells. The same as free DOX, sgc8c-DOX conjugates were cytotoxic to CCRF-CEM cells. More importantly, these conjugates showed excellent selective cytotoxicity, increasing toxicity to target CCRF-CEM cells by 6.7-fold compared to the acute promyelocytic leukemia cell line NB-4. Recently, the same strategy was also adopted by another group to non-covalently link daunorubicin to sgc8, resulting in the specific delivery of daunorubicin to T-cell acute lymphoblastic leukemia cells [55].

In another example, aptamers generated by cell-SELEX were conjugated to a photosensitizer (PS) for the phototherapeutic targeting of tumor cells. Photodynamic therapy (PDT), which has emerged as a promising treatment for cancer, relies on the interaction of excited photosensitizers with ambient oxygen to produce reactive oxygen species (ROS) that kill malignant cells by apoptosis and/or necrosis [56]. However, a key challenge in PDT is the targeted delivery of PS to the cancer site. To address this problem, Mallikaratchy *et al.* conjugated the photosensitizer chlorin e6 (c) to the aptamer TD05 selected against the Burkitt’s lymphoma cell line Ramos [57]. Under illumination, the TD05-Ce6 complex selectively destroyed the targeted Ramos cells by more than 50% over untargeted cells, such as CEM, k562, NB4, and HL60 cell lines.

## 5. Cancer Cell Enrichment and Detection Using Aptamers

Detection of cancer in the early stages can significantly improve survival, but a rare cancer cell in a mixture of normal cells is difficult to find in the early stage of pathogenesis, even by a highly trained pathologist. Therefore, the development of new technologies for effective enrichment and sensitive detection of cancer cells in the early stages will greatly enhance treatment efficacy.

Based on the unique properties of aptamers and gold nanoparticles, we developed a sensitive colorimetric assay for the detection of cancer cells [58]. An aptamer, generated by cell-SELEX, was conjugated to 20-nm gold particles and selectively assembled on the surface of target cells. A significant color change resulted because gold nanoparticles possess strong distance-dependent optical properties, while non-target cells did not show any change in color. The assay is so sensitive that as few as 90 cells/sample could be measured.

By combining aptamers and DNAzyme, Zhu *et al.* developed an aptamer-based colorimetric detection assay for the detection of cancer cells [59]. Aptamers were used as the recognition element to target cancer cells, while peroxidase-active DNAzyme was adopted to produce the colorimetric signals by catalyzing the oxidation of ABTS^2−^ to the colored ABTS^−^. Therefore, by integrating aptamer and DNAzyme, it is possible to detect cancer cells based on the color change of a substrate for DNAzyme. This method requires no expensive apparatus, modification or labeling of DNA chains; therefore, it holds the potential for routine detection of nascent cancer in biopsy specimens.

To detect and capture an extremely low concentration of cancer cells in bodily fluids, an aptamer-modified microfluidic device was designed to enrich cancer cells [60]. The aptamer sgc8 was linked to a biotin moiety at the 3′ end for immobilization within a microfluidic channel, and fluorescein was added at the 5′ end for visualization. These additions did not interfere with the specific selectivity of sgc8 for target cells. By using a microfabricated poly(dimethylsiloxane) (PDMS) device, we obtained >80% capture efficiency with 97% purity of the target cells.

To further detect captured cells, a two-nanoparticle assay with aptamers was developed. Aptamers linked to magnetic nanoparticles were used for target cell extraction, while aptamers linked to fluorescent nanoparticles were simultaneously added for sensitive cell detection [61]. Using 65 nm silica-coated magnetic nanoparticles attached to DNA aptamers, target cells were preferentially extracted from a sample, while only a few control cells were collected. In addition, Ru(bpy) nanoparticle-aptamer conjugates enhanced the fluorescence signal of target cells by more than 100-fold after extraction by magnetic nanoparticles compared to Ru(bpy) dye-labeled cells. Therefore, the simultaneous use of nanoparticle-labeled aptamers for targeted cell detection and selection allows for the rapid and accurate analysis of target cells within a larger non-target population. Furthermore, we have extended this method for the collection and detection of multiple cancer cells using high-affinity aptamers for recognition [62].

## 6. Conclusion

Since its discovery in the early 1990s, aptamer technology has made significant strides. In particular, recent advancements in SELEX technology have been extended from aptamer selection against purified protein to selection against living cells, thus enabling the isolation of aptamers that bind to cell-specific proteins *in situ*. Theses aptamers can be applied to many fields of molecular medicine. Using cell-based aptamers, different cell types can be distinguished without prior knowledge of the target molecules. By integrating other technologies, such as nanotechnology or microfluidic technology, aptamers can be used to enrich and detect diseased cells for clinical diagnosis. Furthermore, the identification of aptamer molecular targets constitutes a novel method of biomarker discovery. Also, based on the high specificity and easy chemical modification of aptamers, cell-specific aptamers can be readily adapted for drug delivery and targeted therapy. Undoubtedly, with the rapid progress of nanotechnology, microchips, microfluidic devices, RNAi and other advanced technologies, cell-SELEX represents an integrated technology that will revolutionize the way we diagnose, treat and prevent disease.

## Figures and Tables

**Table 1:** Target identification mediated by cell-SELEX.

Aptamer	Cell Type	Target	Ref.
GBI-10 (ssDNA)	Glioblastoma cell line U251	tenascin-C	[32]
aptamer III.1 (ssDNA)	Endothelial cell line YPEN-1	pigpen	[37]
TD05 (ssDNA)	Burkitt’s lymphoma cell line Ramos	Ig μ heavy chain	[36]
Sgc8 (ssDNA)	T-cell acute lymphoblastic leukemia CCRF-CEM	PTK7	[35]
A07 (RNA)	Chinese hamster ovary (CHO) cell line expressing recombinant transforming growth factor-βtype III receptor	TGF-βRIII	[38]
D4 (RNA)	NGF-different pheochromocytoma cell line PC12 expressing recombinant MEN2A mutant	MEN2A mutant RET	[39]

## References

[1] Mandal M., Breaker R.R. (2004). Gene regulation by riboswitches. Nat. Rev. Mol. Cell Biol.

[2] Ellington A.D., Szostak J.W. (1990). *In vitro* selection of RNA molecules that bind specific ligands. Nature.

[3] Tuerk C., Gold L. (1990). Systematic evolution of ligands by exponential enrichment: RNA ligands to bacteriophage T4 DNA polymerase. Science.

[4] Gold L. (1995). The SELEX process: A surprising source of therapeutic and diagnostic compounds. Harvey Lect.

[5] Famulok M., Mayer G., Blind M. (2000). Nucleic acid aptamers-from selection *in vitro* to applications *in vivo*. Acc. Chem. Res.

[6] Kurreck J. (2003). Antisense technologies. Improvement through novel chemical modifications. Eur. J. Biochem.

[7] Conrad R., Keranen L.M., Ellington A.D., Newton A.C. (1994). Isozyme-specific inhibition of protein kinase C by RNA aptamers. J. Biol. Chem.

[8] Shoji A., Kuwahara M., Ozaki H., Sawai H. (2007). Modified DNA aptamer that binds the (*R*)-isomer of a thalidomide derivative with high enantioselectivity. J. Am. Chem. Soc.

[9] Ruta J., Ravelet C., Baussanne I., Decout J.L., Peyrin E. (2007). Aptamer-based enantioselective competitive binding assay for the trace enantiomer detection. Anal. Chem.

[10] Farokhzad O.C., Cheng J., Teply B.A., Sherifi I., Jon S., Kantoff P.W., Richie J.P., Langer R. (2006). Targeted nanoparticle-aptamer bioconjugates for cancer chemotherapy *in vivo*. Proc. Natl. Acad. Sci. USA.

[11] Bagalkot V., Farokhzad O.C., Langer R., Jon S. (2006). An aptamer-doxorubicin physical conjugate as a novel targeted drug-delivery platform. Angew. Chem. Int. Ed. Engl.

[12] Chen C.H., Dellamaggiore K.R., Ouellette C.P., Sedano C.D., Lizadjohry M., Chernis G.A., Gonzales M., Baltasar F.E., Fan A.L., Myerowitz R. (2008). Aptamer-based endocytosis of a lysosomal enzyme. Proc. Natl. Acad. Sci. USA.

[13] Tong G.J., Hsiao S.C., Carrico Z.M., Francis M.B. (2009). Viral capsid DNA aptamer conjugates as multivalent cell-targeting vehicles. J. Am. Chem. Soc.

[14] McNamara J.O., Andrechek E.R., Wang Y., Viles K.D., Rempel R.E., Gilboa E., Sullenger B.A., Giangrande P.H. (2006). Cell type-specific delivery of siRNAs with aptamer-siRNA chimeras. Nat. Biotechnol.

[15] Brody E.N., Gold L. (2000). Aptamers as therapeutic and diagnostic agents. J. Biotechnol.

[16] Ferreira C.S., Matthews C.S., Missailidis S. (2006). DNA aptamers that bind to MUC1 tumour marker: Design and characterization of MUC1-binding single-stranded DNA aptamers. Tumour Biol.

[17] Lupold S.E., Hicke B.J., Lin Y., Coffey D.S. (2002). Identification and characterization of nuclease-stabilized RNA molecules that bind human prostate cancer cells via the prostate-specific membrane antigen. Cancer Res.

[18] Gutsaeva D.R., Parkerson J.B., Yerigenahally S.D., Kurz J.C., Schaub R.G., Ikuta T., Head C.A. (2011). Inhibition of cell adhesion by anti-P-selectin aptamer: A new potential therapeutic agent for sickle cell disease. Blood.

[19] Townshend B., Aubry I., Marcellus R.C., Gehring K., Tremblay M.L. (2010). An RNA aptamer that selectively inhibits the enzymatic activity of protein tyrosine phosphatase 1B *in vitro*. ChemBioChem.

[20] Liu Y., Kuan C.T., Mi J., Zhang X., Clary B.M., Bigner D.D., Sullenger B.A. (2009). Aptamers selected against the unglycosylated EGFRvIII ectodomain and delivered intracellularly reduce membrane-bound EGFRvIII and induce apoptosis. Biol. Chem.

[21] Sefah K., Shangguan D., Xiong X., O’Donoghue M.B., Tan W. (2010). Development of DNA aptamers using Cell-SELEX. Nat. Protoc.

[22] Morris K.N., Jensen K.B., Julin C.M., Weil M., Gold L. (1998). High affinity ligands from *in vitro* selection: Complex targets. Proc. Natl. Acad. Sci. USA.

[23] Shangguan D., Li Y., Tang Z., Cao Z.C., Chen H.W., Mallikaratchy P., Sefah K., Yang C.J., Tan W. (2006). Aptamers evolved from live cells as effective molecular probes for cancer study. Proc. Natl. Acad. Sci. USA.

[24] Sefah K., Tang Z.W., Shangguan D.H., Chen H., Lopez-Colon D., Li Y., Parekh P., Martin J., Meng L., Phillips J.A. (2009). Molecular recognition of acute myeloid leukemia using aptamers. Leukemia.

[25] Shangguan D., Meng L., Cao Z.C., Xiao Z., Fang X., Li Y., Cardona D., Witek R.P., Liu C., Tan W. (2008). Identification of liver cancer-specific aptamers using whole live cells. Anal. Chem.

[26] Kunii T., Ogura S., Mie M., Kobatake E. (2011). Selection of DNA aptamers recognizing small cell lung cancer using living cell-SELEX. Analyst.

[27] Chen H.W., Medley C.D., Sefah K., Shangguan D., Tang Z., Meng L., Smith J.E., Tan W. (2008). Molecular recognition of small-cell lung cancer cells using aptamers. ChemMedChem.

[28] Zhao Z., Xu L., Shi X., Tan W., Fang X., Shangguan D. (2009). Recognition of subtype non-small cell lung cancer by DNA aptamers selected from living cells. Analyst.

[29] Van Simaeys D., Lopez-Colon D., Sefah K., Sutphen R., Jimenez E., Tan W (2010). Study of the molecular recognition of aptamers selected through ovarian cancer cell-SELEX. PLoS One.

[30] Santoni V., Molloy M., Rabilloud T. (2000). Membrane proteins and proteomics: Un amour impossible?. Electrophoresis.

[31] Mirza S.P., Halligan B.D., Greene A.S., Olivier M. (2007). Improved method for the analysis of membrane proteins by mass spectrometry. Physiol. Genomics.

[32] Daniels D.A., Chen H., Hicke B.J., Swiderek K.M., Gold L. (2003). A tenascin-C aptamer identified by tumor cell SELEX: Systematic evolution of ligands by exponential enrichment. Proc. Natl. Acad. Sci. USA.

[33] Orend G., Chiquet-Ehrismann R. (2006). Tenascin-C induced signaling in cancer. Cancer Lett.

[34] Shangguan D., Li Y., Tang Z., Cao Z.C., Chen H.W., Mallikaratchy P., Sefah K., Yang C.J., Tan W. (2006). From the cover: Aptamers evolved from live cells as effective molecular probes for cancer study. Proc. Natl. Acad. Sci. USA.

[35] Shangguan D., Cao Z., Meng L., Mallikaratchy P., Sefah K., Wang H., Li Y., Tan W. (2008). Cell-specific aptamer probes for membrane protein elucidation in cancer cells. J. Proteome Res.

[36] Mallikaratchy P., Tang Z., Kwame S., Meng L., Shangguan D., Tan W. (2007). Aptamer directly evolved from live cells recognizes membrane bound immunoglobin heavy mu chain in Burkitt’s lymphoma cells. Mol. Cell. Proteomics.

[37] Blank M., Weinschenk T., Priemer M., Schluesener H. (2001). Systematic evolution of a DNA aptamer binding to rat brain tumor microvessels. selective targeting of endothelial regulatory protein pigpen. J. Biol. Chem.

[38] Ohuchi S.P., Ohtsu T., Nakamura Y. (2006). Selection of RNA aptamers against recombinant transforming growth factor-beta type III receptor displayed on cell surface. Biochimie.

[39] Cerchia L., Duconge F., Pestourie C., Boulay J., Aissouni Y., Gombert K., Tavitian B., de Franciscis V., Libri D (2005). Neutralizing aptamers from whole-cell SELEX inhibit the RET receptor tyrosine kinase. PLoS Biol.

[40] Zueva E., Rubio L.I., Ducongé F., Tavitian B. (2011). Metastasis-focused cell-based SELEX generates aptamers inhibiting cell migration and invasion. Int. J. Cancer.

[41] Cerchia L., Esposito C.L., Jacobs A.H., Tavitian B., de Franciscis V (2009). Differential SELEX in human glioma cell lines. PLoS One.

[42] Boltz A., Piater B., Toleikis L., Guenther R., Kolmar H., Hock B. (2011). Bi-specific aptamers mediating tumor cell lysis. J. Biol. Chem.

[43] Ye M., Hu D., Tu L., Zhou X., Lu F., Wen B., Wu W., Lin Y., Zhou Z., Qu J. (2008). Involvement of PI3K/Akt signaling pathway in hepatocyte growth factor-induced migration of uveal melanoma cells. Invest. Ophthalmol. Vis. Sci.

[44] Davis M.I., Bennett M.J., Thomas L.M., Bjorkman P.J. (2005). Crystal structure of prostate-specific membrane antigen, a tumor marker and peptidase. Proc. Natl. Acad. Sci. USA.

[45] Liu H., Rajasekaran A.K., Moy P., Xia Y., Kim S., Navarro V., Rahmati R., Bander N.H. (1998). Constitutive and antibody-induced internalization of prostate-specific membrane antigen. Cancer Res.

[46] Dhar S., Gu F.X., Langer R., Farokhzad O.C., Lippard S.J. (2008). Targeted delivery of cisplatin to prostate cancer cells by aptamer functionalized Pt(IV) prodrug-PLGA-PEG nanoparticles. Proc. Natl. Acad. Sci. USA.

[47] Wang A.Z., Bagalkot V., Vasilliou C.C., Gu F., Alexis F., Zhang L., Shaikh M., Yuet K., Cima M.J., Langer R. (2008). Superparamagnetic iron oxide nanoparticle-aptamer bioconjugates for combined prostate cancer imaging and therapy. ChemMedChem.

[48] Bagalkot V., Zhang L., Levy-Nissenbaum E., Jon S., Kantoff P.W., Langer R., Farokhzad O.C. (2007). Quantum dot-aptamer conjugates for synchronous cancer imaging, therapy, and sensing of drug delivery based on bi-fluorescence resonance energy transfer. Nano Lett.

[49] Chu T.C., Marks J.W., Lavery L.A., Faulkner S., Rosenblum M.G., Ellington A.D., Levy M. (2006). Aptamer: Toxin conjugates that specifically target prostate tumor cells. Cancer Res.

[50] Wullner U., Neef I., Eller A., Kleines M., Tur M.K., Barth S. (2008). Cell-specific induction of apoptosis by rationally designed bivalent aptamer-siRNA transcripts silencing eukaryotic elongation factor 2. Curr. Cancer Drug Targets.

[51] Dassie J.P., Liu X.Y., Thomas G.S., Whitaker R.M., Thiel K.W., Stockdale K.R., Meyerholz D.K., McCaffrey A.P., McNamara J.O., Giangrande P.H. (2009). Systemic administration of optimized aptamer-siRNA chimeras promotes regression of PSMA-expressing tumors. Nat. Biotechnol.

[52] Xiao Z., Shangguan D., Cao Z., Fang X., Tan W. (2008). Cell-specific internalization study of an aptamer from whole cell selection. Chemistry.

[53] Huang Y.-F., Shangguan D., Liu H., Phillips J.A., Zhang X., Chen Y., Tan W. (2009). Molecular assembly of an aptamer-drug conjugate for targeted drug delivery to tumor cells. ChemBioChem.

[54] Shangguan D., Tang Z., Mallikaratchy P., Xiao Z., Tan W. (2007). Optimization and modifications of aptamers selected from live cancer cell lines. ChemBioChem.

[55] Taghdisi S.M., Abnous K., Mosaffa F., Behravan J. (2010). Targeted delivery of daunorubicin to T-cell acute lymphoblastic leukemia by aptamer. J. Drug Target.

[56] Castano A.P., Mroz P., Hamblin M.R. (2006). Photodynamic therapy and anti-tumour immunity. Nat. Rev. Cancer.

[57] Mallikaratchy P., Tang Z., Tan W. (2008). Cell specific aptamer–photosensitizer conjugates as a molecular tool in photodynamic therapy. ChemMedChem.

[58] Medley C.D., Smith J.E., Tang Z., Wu Y., Bamrungsap S., Tan W. (2008). Gold nanoparticle-based colorimetric assay for the direct detection of cancerous cells. Anal. Chem.

[59] Zhu X., Cao Y., Liang Z., Li G. (2010). Aptamer-based and DNAzyme-linked colorimetric detection of cancer cells. Protein Cell.

[60] Phillips J.A., Xu Y., Xia Z., Fan Z.H., Tan W. (2009). Enrichment of cancer cells using aptamers immobilized on a microfluidic channel. Anal. Chem.

[61] Herr J.K., Smith J.E., Medley C.D., Shangguan D., Tan W. (2006). Aptamer-conjugated nanoparticles for selective collection and detection of cancer cells. Anal. Chem.

[62] Smith J.E., Medley C.D., Tang Z., Shangguan D., Lofton C., Tan W. (2007). Aptamer-conjugated nanoparticles for the collection and detection of multiple cancer cells. Anal. Chem.

